# Modulation of NKT Cell Development by B7-CD28 Interaction: An Expanding Horizon for Costimulation

**DOI:** 10.1371/journal.pone.0002703

**Published:** 2008-07-16

**Authors:** Xincheng Zheng, Huiming Zhang, Lijie Yin, Chyung-Ru Wang, Yang Liu, Pan Zheng

**Affiliations:** 1 Department of Surgery, Comprehensive Cancer Center, Program of Molecular Mechanism of Diseases, University of Michigan, Ann Arbor, Michigan, United States of America; 2 OncoImmune Ltd., Columbus, Ohio, United States of America; 3 Department of Pathology, University of Chicago, Chicago, Illinois, United States of America; New York University School of Medicine, United States of America

## Abstract

It has been demonstrated that the development of NKT cells requires CD1d. The contribution of costimulatory molecules in this process has not been studied. Here we show that in mice with targeted mutations of B7-1/2 and CD28, the TCRβ^+^α-Galcer/CD1d ^+^ (iVα14 NKT) subset is significantly reduced in the thymus, spleen and liver. This is mainly due to decreased cell proliferation; although increased cell death in the thymi of CD28-deficient mice was also observed. Moreover, in the B7-1/2- and CD28-deficient mice, we found a decreased percentage of the CD4^−^NK1.1^+^ subset and a correspondingly increased portion of the CD4^+^NK1.1^−^ subset. In addition, the mice with a targeted mutation of either B7 or CD28 had a reduced susceptibility to Con A induced hepatitis, which is known to be mediated by NKT cells. Our results demonstrate that the development, maturation and function of NKT cell are modulated by the costimulatory pathway and thus expand the horizon of costimulation into NKT, which is widely viewed as a bridge between innate and adaptive immunity. As such, costimulation may modulate all major branches of cell-mediated immunity, including T cells, NK cells and NKT cells.

## Introduction

The notion of costimulation was proposed as the signal 2 for the activation of T lymphocytes [1]–[3]. Over the last two decades, B7 and CD28 families have emerged as the prototypical costimulatory ligands and receptors respectively [4]. Although the main emphasis in the field has been on the role of these interactions in the activation of T cells, accumulating data have demonstrated that this pathway also modulates effector function of T cells in cancer rejection [5]–[7] and autoimmune diseases [8], [9]. Moreover, clear evidence has been reported that support an important role for costimulation in the development of T cells [10]. Thus, the costimulatory pathway can in fact function through the whole life-span of T cells. Another interesting development in the field is that the costimulatory molecules ascribed to T cell immunity also modulate innate NK cell immune responses, including both expansion and effector function of NK cells [11]. An interesting issue is whether costimulation modulates the NKT subset, which is generally viewed as the bridge between innate and adaptive immunity.

NKT cells are a unique subset of T cells expressing both TCR and NK cell markers [12]–[16]. However, unlike NK cells, NKT cells mainly develop in the thymus. In contrast to conventional T cells, NKT cells respond to antigen presented by the nonpolymorphic major histocompatibility complex (MHC) Class I-like molecule CD1d [14]–[16]. The TCR of a major subset of NKT cells is composed almost exclusively of Vα14/Jα18 in the mouse and Vα24/Jα18 in human. In mice the TCR receptor α chain is paired with the Vβ8.2, Vβ7, or Vβ2 TCRβ chain. This subset recognize glycolipids presented by CD1d molecules [15], [16]. Almost all NKT cells are either CD4^+^ or CD4^−^CD8^−^ (DN). Upon stimulation through their TCR, the NKT cells rapidly produce substantial amounts of cytokines, such as IL-4 and IFN-γ [17], [18]. Emerging data demonstrate that it is involved in immunity against infection [19] and tumor [20] and in autoimmune disease [21] by either direct cytotoxicity or secretion of several cytokines.

The developmental relationship between NKT and conventional T cells has been controversial. Two models have been proposed for NKT cell development [22], [23]. NKT cells might derive from a distinct precursor that undergoes lineage commitment independently of TCR-ligand interactions (pre-committed model). Alternatively, they may develop as a byproduct of conventional T cell development at a certain stage, depending on the ligand they recognize (TCR-instruction model). Recently, by intrathymic injection, Gapin et al. showed that NKT cells can be produced in the thymus from CD4+CD8+ DP thymocyte [24]. An intermediate, semi-mature CD4^+^NK1.1^−^ stage has been proposed before NKT cells finally develop into NK1.1^+^ cells that are either CD4^+^ or double negative [25]–[27]. Moreover, the positive and negative selection of NKT cells rely on CD1d being presented by hematopoietic derived DP thymocyte or dendritic cells, respectively [24], [28], but not those presented by thymic epithelial cells. The details of this selective process are still largely unknown. A largely unresolved issue is whether the B7-CD28 interaction is involved in the development of NKT cells. In order to address this question, we compared the development of NKT cells in WT mice to that in mice with targeted mutations of B7-1/B7-2 or CD28. Our results demonstrate that the development and function of NKT cells are subject to modulation by costimulatory pathways.

## Results

### The reduced TCR β^+^NK1.1^+^ NKT cells in B7-1/2 and CD28 deficient mice

**Figure 1:**
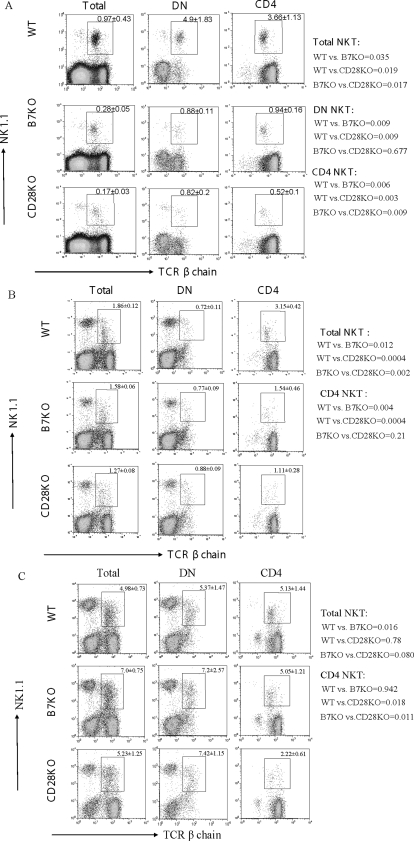
Roles for B7-1/2-CD28 interaction in the accumulation of TCR β^+^NK1.1^+^ NKT cells in the thymus (A), spleen (B) and liver (C). B7KO, CD28KO and WT C57BL/6 mice were sacrificed at 8-week of age. Total lymphocytes (left column) from viable cells, CD4^−^CD8^−^ DN lymphocytes (middle column) and CD4^+^ lymphocytes (right column) were analyzed for the expression of TCRβ and NK1.1. One representative profile from each group is presented. The numbers in the panels are percentages (Mean±SD) of gated NKT cells (n = 9). The numbers shown on the side of figures are *p* value between two indicated groups.

In order to evaluate the role for costimulatory molecules on NKT development, we compared the percentages of NKT cells in the central and peripheral lymphoid organs of B7-1/2, CD28 mutant mice with those of NKT cells in the same organs of age and sex- matched WT mice. In this study, the total NKT cells were defined as NK1.1^+^TCRβ^+^ cells. As shown in Fig. 1A, TCR β^+^NK1.1^+^ cells comprise about 1% of the total thymocytes in WT mice. After dividing the thymocytes into DN, DP and SP subsets, it was clear that NKT cells were largely absent from the DP and CD4^−^CD8^+^ subsets, as others have reported [15]. About 5% of DN and 4% of CD4^+^CD8^−^ thymocytes expressed the NKT marker in WT mice. A 3–5 fold reduction of the NKT cells was observed in total thymocytes and DN and CD4 subsets from both B7-1/2- and CD28- deficient mice. Thus, B7-CD28 interaction is required for the development of NKT in the thymus.

We also compared the amounts of NKT cells in the spleens and livers of mice deficient for B7-1/2 and CD28 with those of WT mice. As shown in Fig. 1B, total TCR β^+^NK1.1^+^ cells in the spleen were decreased somewhat as a result of targeted mutation of B7 and CD28. Among the CD4^+^ splenocytes, the NKT subset is reduced by more than 2-fold. However, the reduction in DN NKT was marginal. The liver is known to be populated with a high number of NKT cells [29]–[31]. However, except for the reduction in NKT among the CD4^+^CD8^−^ subset in the CD28 deficient mice, targeted mutation of B7-CD28 did not have a significant impact on the number of TCR β^+^NK1.1^+^ NKT cells (Fig. 1C).

**Figure 2:**
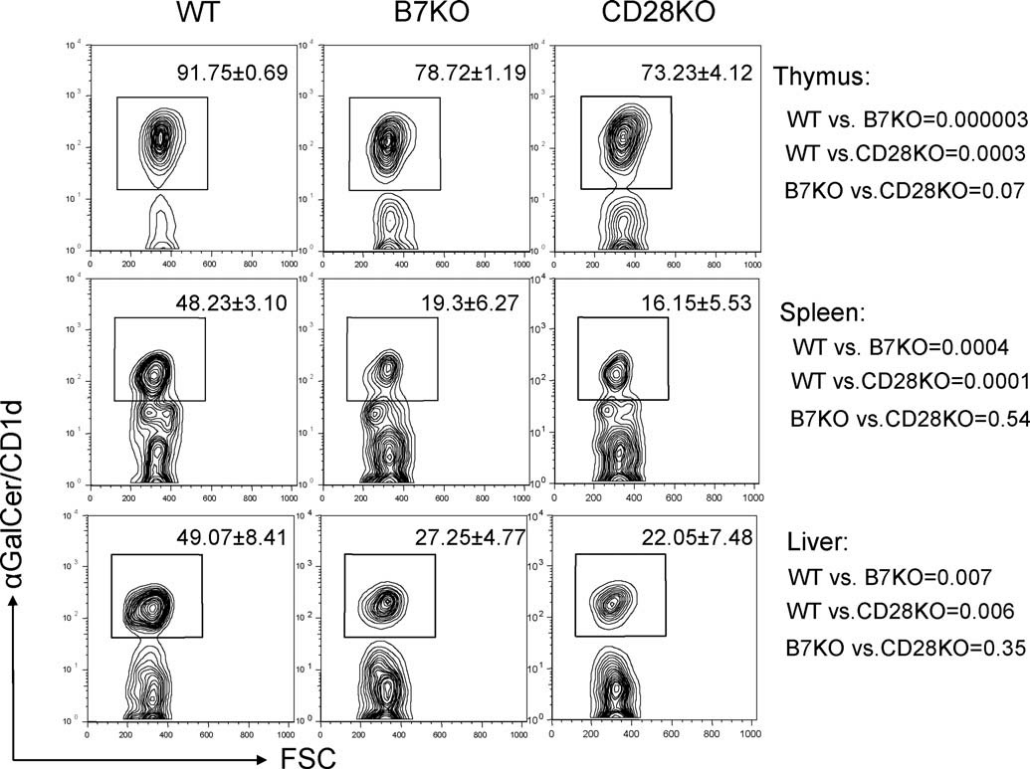
Proportions of iVα14 NKT cells among TCR β^+^NK1.1^+^ cells isolated from the thymus, spleen and liver of WT, B7-1/2 and CD28 deficient mice. Total lymphocytes from the thymus (left), spleen (middle) and liver (right) of 8-week old WT (top), B7-1/2- (center) and CD28- (bottom) deficient mice were stained with TCRβ, NK1.1 and α-GalCer/CD1d tetramer. The numbers represent the percentage (Mean±SD, n = 9) of α-GalCer/CD1d^+^ cells in the TCR β^+^NK1.1^+^ population. One representative profile from each group is shown here. The numbers shown on the side of figures are *p* value between two indicated groups.

Most of the NKT cells recognize glycolipids presented by MHC class I-like molecule CD1d, and are referred to as iVα14 NKT cells for being CD1d-restricted NKT bearing the invariant Vα14 rearrangement [32], [33]. Although the identity of the endogenous antigen presented by CD1d is still unclear, a synthetic glycolipid antigen derived from marine sponges called alpha-galactosyl ceramide (α-GalCer) can be recognized by NKT and potently stimulate NKT cells via TCR when presented by CD1d [34]. Recently, the development of αGalCer/CD-1d tetramer, which recognizes invariant Vα14–Jα281 TCR, provides another powerful tool to identify iVα14 NKT cells. αGalCer/CD-1d tetramer positive cells comprised about 80% of NK1.1^+^TCRβ^+^ cells [35], [36]. Those αGalCer/CD-1d tetramer negative cells were identified as different subsets of NKT cells, which are preferentially localized in spleen and bone marrow and are predominantly of the CD8^+^ (or DN) phenotype [23]. In this study, we used the tetramer to further characterize the TCR β^+^NK1.1^+^ cells in B7-1/2 and CD28 deficient mice. As shown in Fig. 2, about 90% of the TCR β^+^NK1.1^+^ cells in WT thymus bind to α-GalCer-CD1d tetramer. In B7- and CD28- deficient thymus only about 75% of the TCR β^+^NK1.1^+^ NKT bind to the tetramer. The reduction of the α-GalCer-CD1d tetramer binding cells in the spleen and liver is more severe in the mutant mice. Whereas in WT mice, there were close to 50% of spleen and liver derived TCR β^+^NK1.1^+^ NKT cells that bound to the tetramer, and less than 20% of TCR β^+^NK1.1^+^ NKT cells in mutant mice showed similar specificity in the spleen, and the tetramer binding cells were reduced by 2-fold in the liver of the mutant mice.

### B7-CD28 interaction promotes expansion of NK1.1 on the iVα14 NKT cells

**Figure 3:**
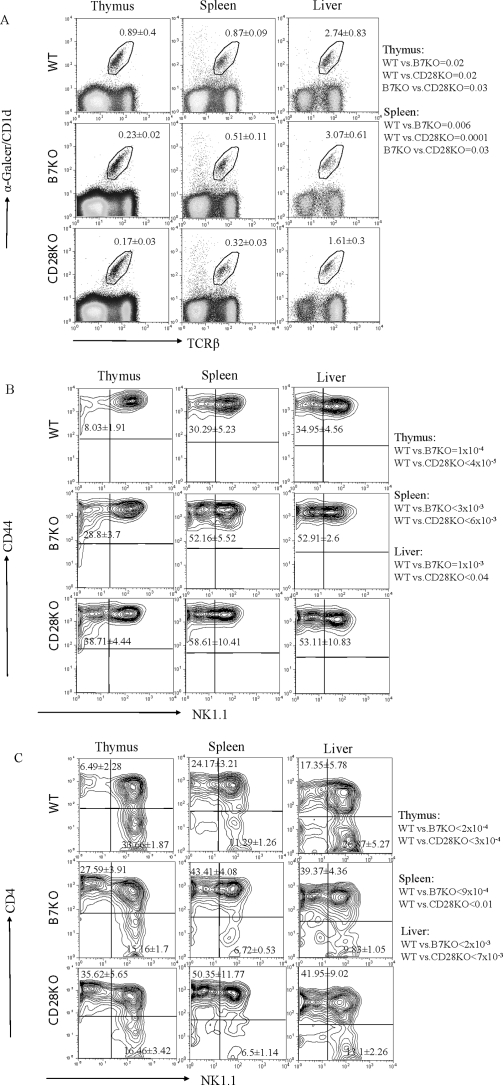
Quantitative and phenotypic variation of iVα14 NKT cells in the thymus, spleen and liver of WT, B7-1/2 and CD28 deficient mice. Total lymphocytes from the thymus (left), spleen (middle) and liver (right) of 8-week old WT (top), B7-1/2 (center) and CD28 (bottom) deficient mice were analyzed. A. The quantification of iVα14 NKT cells. The numbers represent the percentage (Mean±SD, n = 9) of iVα14 NKT cells. B. The expression of CD44 and NK1.1 among the gated iVα14 NKT cells. The numbers represent the percentage (Mean±SD) of NK1.1^−^ iVα14 NKT cells. C. The expression of CD4 and NK1.1 from gated iVα14 NKT cells. The numbers represent the percentage (Mean±SD) of NK1.1^−^CD4^+^ (upper left) and NK1.1^+^CD4^−^ (lower right) iVα14 NKT cells. One representative profile from each group is shown here. The numbers shown on the side of figures are *p* value between two indicated groups.

To further characterize the effect of costimulatory molecules on iVα14 NKT cell development and activation, we analyzed the number of cells as well as NK1.1, CD44 and CD4 expression among iVα14 NKT cells. As shown in Fig. 3A, the number of iVα14 NKT cells was significantly reduced in total lymphocytes from both the thymus and spleen of B7-1/2 deficient mice. Again, a qualitatively similar but quantitatively more significant reduction was detected in CD28 deficient mice. While a small reduction was observed in the liver of B7- deficient mice, no reduction of iVα14 NKT was observed in the liver of CD28-deficient mice. Thus, the optimal production of iVα14 NKT cells in the thymus and their accumulation in the spleens require B7-CD28 interaction.

Recent studies have demonstrated that in normal mice the NK1.1^+^ iVα14 NKT cells are developed from NK1.1^−^ iVα14 NKT cells. Interestingly, in B7-1/2 and CD28 deficient mice, the percentage of NK1.1^+^ subsets among iVα14 NKT cells was significantly reduced, while the NK1.1^−^ subsets were significantly increased by 4-fold in the thymus and 2-fold in the spleen and liver (Fig. 3B). The iVα14 NKT cells could be further divided into three major subsets, NK1.1^−^CD4^+^, NK1.1^+^CD4^+^ and NK1.1^+^CD4^−^. Compared to wild type mice, targeted mutation of both B7 and CD28 caused a significant reduction in the NK1.1^−^CD4^+^ subset and a corresponding increase in the NK1.1^+^CD4^−^ subset (Fig. 3C). If the reduction of iVα14 NKT cells from total lymphocytes is considered, the reduction of the NK1.1^+^ iVα14 NKT subset seen in the mutant mice is even more significant when compared to WT mice, and the seemingly observed increase in the NK1.1^−^iVα14 NKT subset is actually unchanged (Table 1).

**Table 1:** Different iVα14 NKT subsets in total lymphocytes of thymus, spleen and liver in WT, B7-1/2 and CD28 deficient mice.

		WT	B7KO	CD28KO
Thymus	NK1.1^−^CD4^+^	0.05±0.02	0.06±0.01	0.06±0.01
	NK1.1^+^CD4^+^	0.52±0.23	0.13±0.02*	0.03±0.01*
	NK1.1^+^CD4^−^	0.3±0.15	0.03±0.01*	0.03±0.01*
Spleen	NK1.1^−^CD4^+^	0.21±0.04	0.22±0.05	0.16±0.03
	NK1.1^+^CD4^+^	0.53±0.07	0.23±0.06**	0.13±0.05***
	NK1.1^+^CD4^−^	0.1±0.01	0.03±0.01***	0.02±0.01***
Liver	NK1.1^−^CD4^+^	0.48±0.26	1.21±0.29*	0.68±0.21
	NK1.1^+^CD4^+^	1.34±0.44	1.46±0.33	0.64±0.19*
	NK1.1^+^CD4^−^	0.72±0.26	0.3±0.05*	0.21±0.05*

Lymphocytes from thymus, spleen and liver of age and sex-matched WT, B7-1(-/-)/B7-2(-/-) or CD28(-/-) mice were analyzed by flow cytometry. The iVα14 NKT cells were identified based on expressing TCRβ and binding to the α-Galcer/CD1d tetramer. Data shown are means and SD of the percentage of given subsets in total viable lymphocytes (n = 4). Similar data were obtained in another experiment involving 5 mice per group.

* P<0.05,

** P<0.01,

*** P<0.001.

### Normal generation of immature iVα14 NKT cells in young B7- and CD28-deficient mice

**Figure 4:**
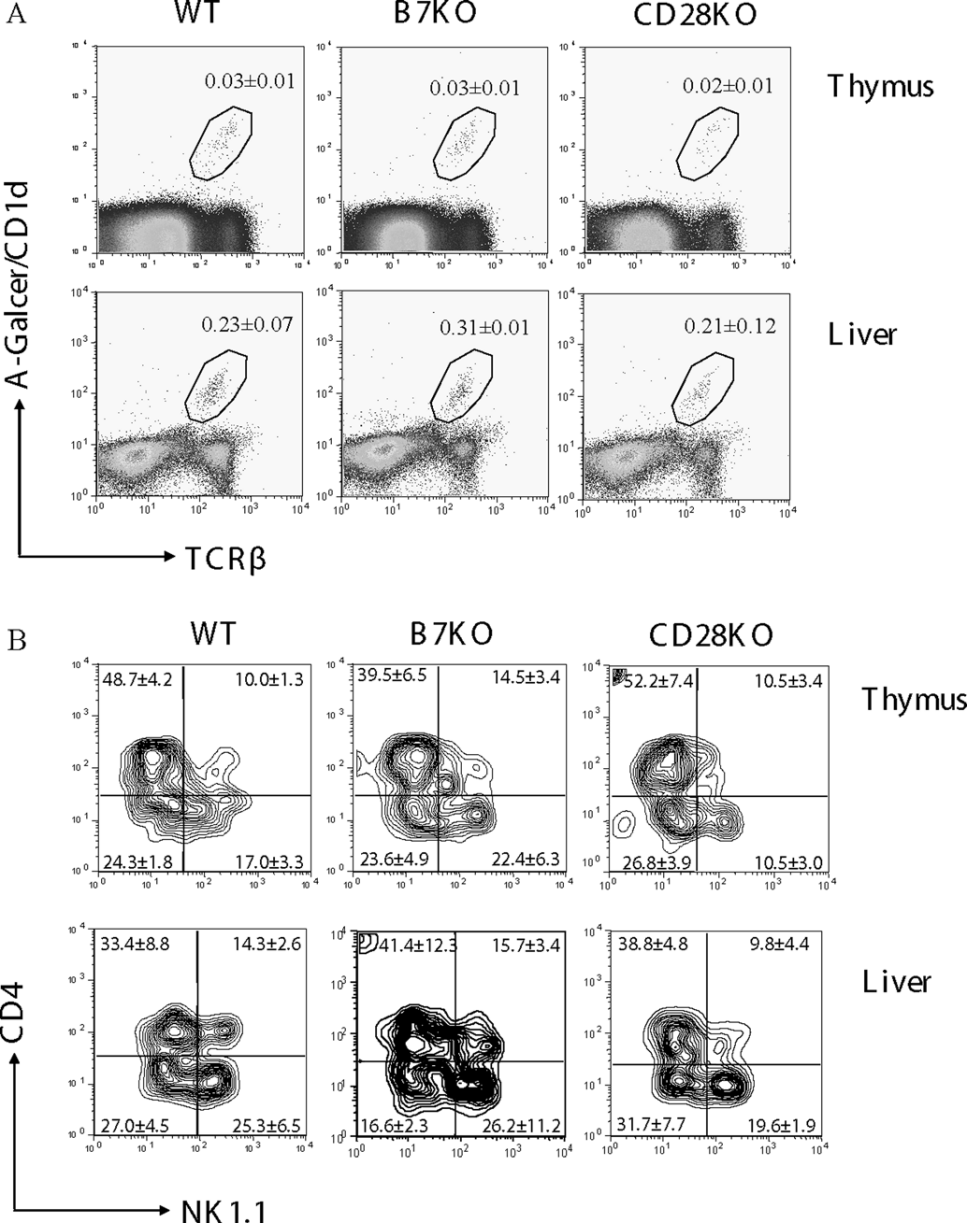
B7- and CD28-deficiency does not affect the generation of immature iVα14 NKT cells in young mice. Total lymphocytes from the thymus (upper panel) and liver (lower panel) of 8-day old WT (left), B7-1/2- (middle) and CD28- (right) deficient mice were analyzed. The numbers represent the percentage (Mean±SD, n = 4) of iVα14 NKT cells (A) and the percentage (Mean±SD, n = 4) of individual subsets from gated iVα14 NKT cells (B). One representative profile from each group is shown.

In the thymus, a low number of iVα14 NKT cells can be detected by α-GalCer/CD1d tetramer as early as 5 days after birth [16], [27]. Thereafter, both the percentage and the absolute number of iVα14 NKT cells increase reaching a peak level at around 6 weeks [24], [27]. To determine if B7-CD28 interaction is required for the generation of iVα14 NKT cells in young mice, we analyzed the number and phenotypes of iVα14 NKT cells in the thymi and livers of 8-day old mice. As shown in Fig. 4A, the total percents of tetramer binding cells in WT and mutant mice were only around 0.03% in the thymus and 0.2% in the liver. Both the number and the pattern of NK1.1 and CD4 are consistent with that previously observed in WT mice [27]. Pellicci et al. also demonstrated that at day11 of fetal thymus organ culture FTOC, α-GalCer/CD1d tetramer NKT cells only account for less than 5% of total NK1.1^+^TCRβ^+^ cells [27]. Therefore, the percentage of total NKT cells at 8 days of age should be more than the above numbers. In all strains of mice, the majority of iVα14 NKT cells were immature as they did not yet express NK1.1 (Fig. 4B). Thus, B7-CD28 interaction appears selectively involved in the maturation of NKT cells in adult mice.

### B7-CD28 interaction promotes proliferation of NKT cells

**Figure 5:**
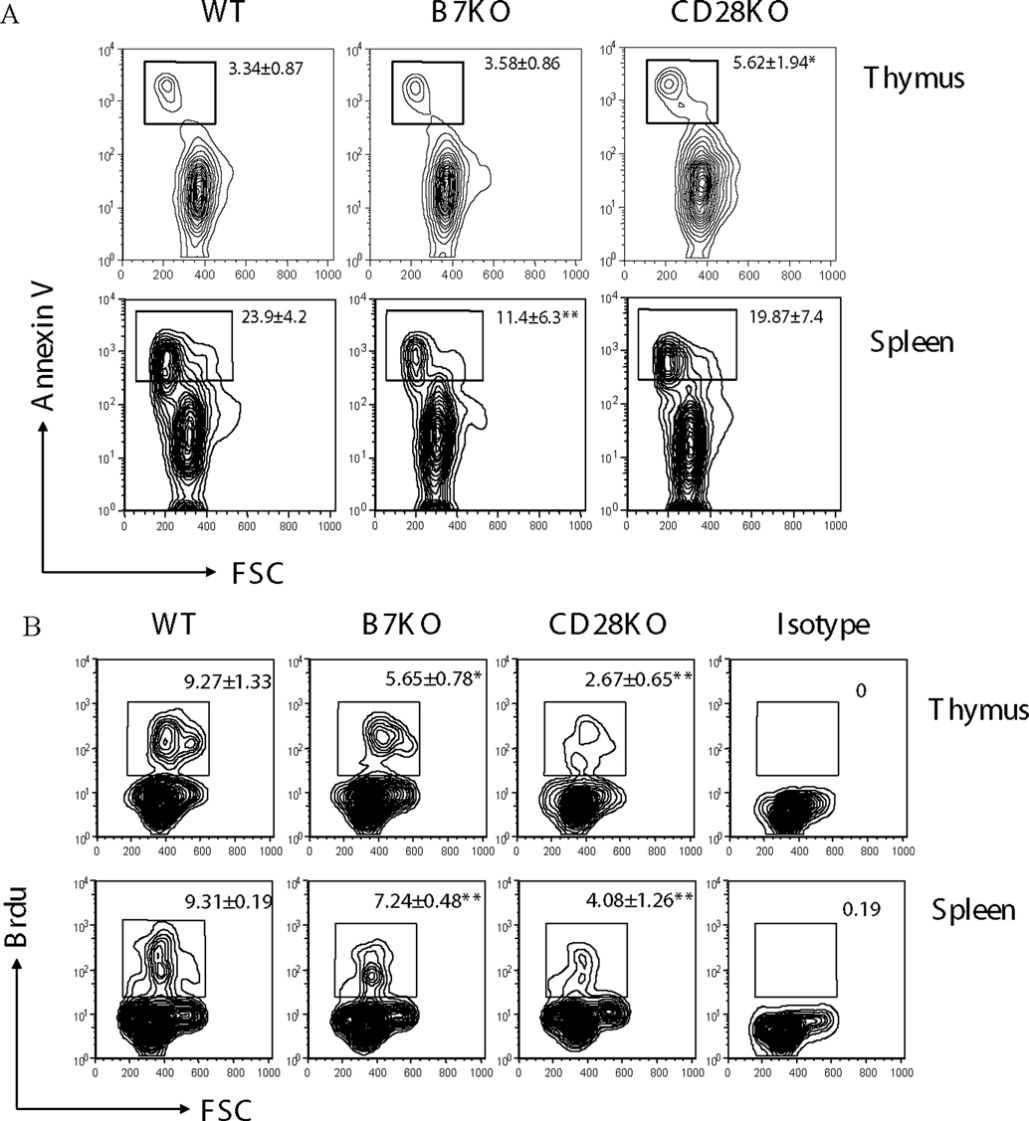
B7-CD28 interaction promotes proliferation but not survival of NKT cells. 5–7-week old WT, B7KO, CD28KO mice received intravenous injection of BrdU. The lymphocytes from the thymus (top) and spleen (bottom) were harvested 4 hours later and stained with antibodies for TCRβ, NK1.1 and BrdU or with Annexin V. A. NKT cells that underwent apoptosis. The numbers represent the percentages (Mean±SD) of apoptotic cells among TCRβ^+^NK1.1^+^ cells and summarizing data from 2 independent experiments involving a total of 6 mice in the WT and CD28 mutant group, and 3 in B7-1/2 mutant mice. B. NKT cells undergo proliferation *in vivo* as revealed by incorporation of BrdU. The numbers in the panels indicate means and SD (n = 3) of the percentages of NKT cells that have incorporated BrdU in the thymus. FITC conjugated mouse IgG_1_, κ was used as isotype control (right). One representative profile from each group is shown.

The reduced cell number can be explained by increased cell death and/or decreased cell proliferation in the organ. Therefore, we first tested whether B7 and CD28 deficiencies affect the survival of iVα14 NKT cells. We compared the number of cells undergoing programmed cell death in WT, B7-1/2- and CD28-deficient mice by staining with Annexin V. Since the reduction of iVα14 NKT cells is limited to the NK1.1^+^ subset, we analyzed apoptosis of TCR^+^NK1.1^+^ NKT cells. As shown in Fig. 5A, in WT mice, there were about 3% Annexin V^+^ cells in the thymic TCR^+^NK1.1^+^ cells and 20% in the same subsets from the spleen. While the same amount of Annexin V^+^ cells was detected in the thymus of B7-1/2 deficient mice, CD28-deficient mice had an increase in Annexin V^+^ cells in the thymus, but not in the spleen. Thus, while a contribution of CD28 in the survival of NKT cells in the thymus cannot be ruled out, decreased survival does not explain the defect in the development of iVα14 NKT cells.

To investigate whether B7- and CD28-deficiencies affect the proliferation of NKT cells, we pulsed WT, B7- and CD28- deficient mice with BrdU and measured DNA synthesis in TCR^+^NK1.1^+^ by flow cytometry. As shown in Fig. 5B, about 10% of the TCR^+^NK1.1^+^ cells were undergoing proliferation in WT mice during the 4h period, the percentages of BrdU^+^ cells were reduced by 2 fold in B7-1/2 deficient mice and 4 fold in CD28 deficient mice in the thymus. A significant although less pronounced reduction was observed in the spleens of the mutant mice. The more significant inhibition of cell proliferation and somewhat increased cell death of NKT cells in the thymus might explain the more severe reduction of NKT cells in mice with targeted mutations of CD28.

### The defective NKT function in costimulatory molecule deficient mice

**Figure 6:**
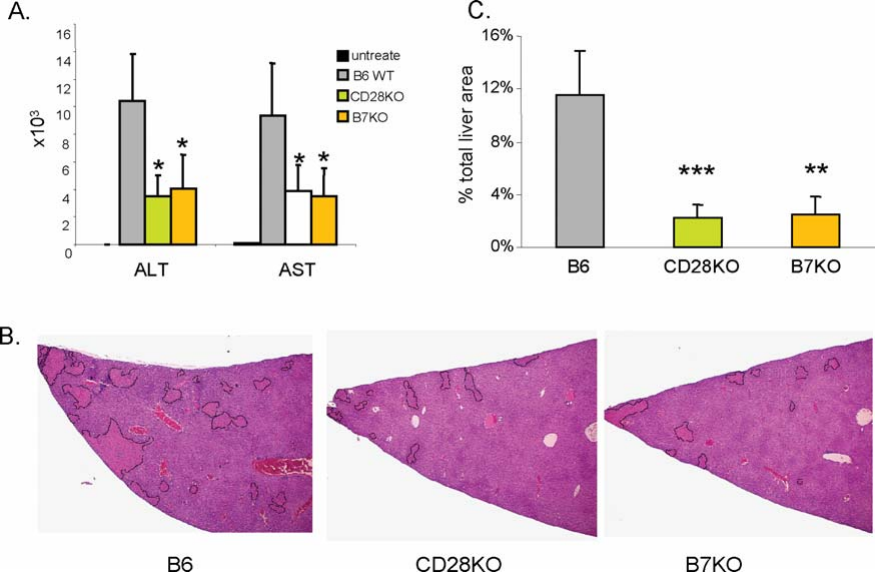
Con A induced hepatitis is alleviated in B7- and CD28- deficient mice. Eight-week old B7KO, CD28KO and C57BL/6 mice received intravenous injection of Con A at a dose of 20 mg/kg. Sixteen hours later, serum and liver tissues from individual mice were harvested. A. Serum ALT and AST levels. B. One representative section from each group showing the pattern of liver injury. C. Average percentages of area with injuries from 3–4 sections per mouse and 4–5 mice per group.

Several publications have demonstrated the contribution of liver NKT cells to a Con A induced murine hepatitis model [37], [38]. To explore if there is possible defective NKT function due to the reduction of iVα14 NKT cells in B7 and CD28 deficient mice, we injected Con A into B7-1/2, CD28 deficient mice and WT mice and measured serum AST and ALT levels for liver damage. The liver sections were also examined after H&E staining. As shown in Fig. 6, severe liver damage was induced in WT mice as revealed by high serum AST and ALT levels and necrosis of hepatocytes. In the B7- and CD28- deficient mice, serum AST and ALT levels were reduced by more than 2-fold in mutant mice as compared with WT mice (Fig. 6A). Corresponding to this we also observed a 4–5-fold reduction in the necrotic area of the livers from B7- and CD28- deficient mice (Fig. 6B and C).

### Reduced level of Con A induced IFN-γ production in B7-1/2 deficient mice

**Figure 7:**
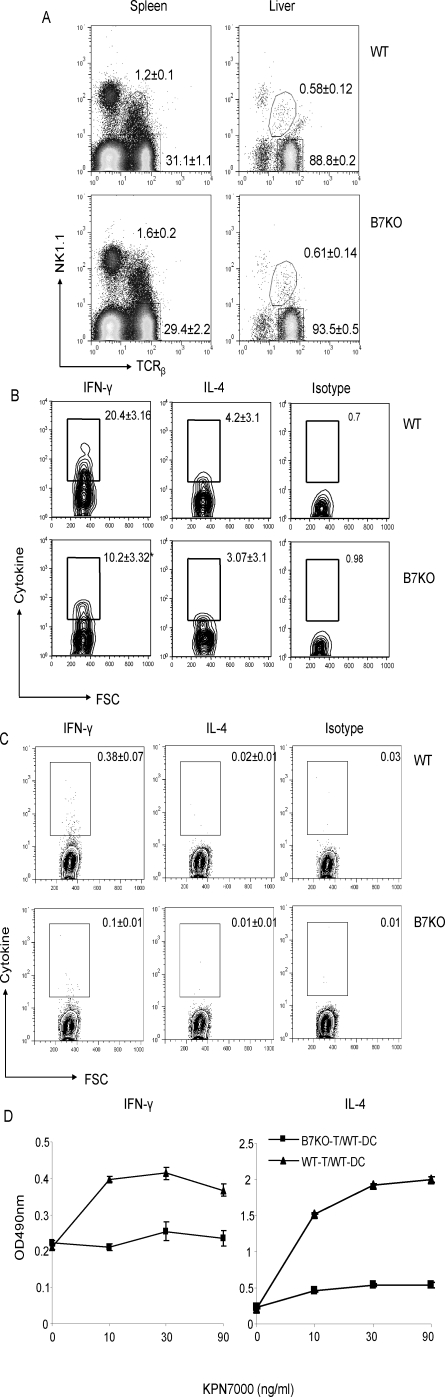
The reduction of IFN-γ production among TCR β^+^NK1.1^+^ cells isolated from the liver of B7-1/2 deficient mice. Eight-week old WT (top) and B7-1/2- (bottom) deficient mice received intravenous injection of Con A (25mg/kg). The mice were sacrificed six hours later. After perfusion the total number of mononuclear cells from the liver and spleen were stained with TCRβ, NK1.1, anti-mouse IFN-γ or IL-4 mAbs, or fluorescence conjugated isotype control. A. The percentage of total NKT cells (NK1.1^+^TCR β^+^) and conventional T cells (NK1.1^+^TCR β^+^) in the spleen and liver from Non-treated (left) and ConA treated mice (right). B. The percentage of cytokine producing cells among the NK1.1^+^TCR β^+^ population. C. The percentage of cytokine producing cells among the NK1.1^−^TCR β^+^ population. The numbers represent the percentage (Mean±SD) of one representative profile from each group is shown. The experiment was repeated twice; 7–8 mice were included in each group. D. Cytokine secretion of purified CD4 T cells from WT and B7KO mice stimulated by KRN7000. The numbers represent the profile of purified CD4 T cells from 3 mice in each group. The experiment was repeated twice.

Con A induced hepatitis is a multiple factors disease process. Both Th1 type cytokine IFN-γ [39]–[41] and Th2 type cytokine IL-4 [37], [38], [42] are crucially involved in the induction of NKT cell-mediated liver specific inflammation. Moreover, other immunoregulatory cytokines, such as IL-5 [42], [43] and IL-6 [44], had also been demonstrated to mediate the pathogenesis of this disease. We investigated whether the reduced susceptibility to Con A induced hepatitis in B7-1/2 deficient mice is due to defective cytokine production. *Ex vivo* mononuclear cells were analyzed at 6 hours after Con A treatment *in vivo*. As shown in Fig. 7A, compared with Non-treated mice, the percentage of total NKT cells was reduced almost 10-fold in in the livers of ConA treated WT and B7KO mice. Correspondingly, the conventional T cells, which were identified by NK1.1^−^TCRβ^+^ cells are relatively increased. Interestingly, those NKT cells reside in the spleen are not affected. Comparable numbers of total NKT cells are detected before and after ConA treatment in the spleens of both strains. This further demonstrated that NKT cells in the liver play a major role inducing acute hepatitis. In addition, as shown in Fig. 7B, in WT mice, about 20% of he liver NKT cells produced IFN-γ and 4% of the NKT cells produced high levels of IL-4. The amount of the same level of IFN-γ producing cells was reduced by 50% in B7-1/2 deficient mice, while the amount of high levels of IL-4 producing cells was unchanged. It has been shown that the peak of IL-4 production by NKT cells is earlier than IFN- γ [45], [46]. We also confirmed that B7-1/2 deficient NKT cells in the spleen produced less IL-4 compared with WT mice at an earlier time point (unpublished observation). However, both IFN-γ and IL-4 cytokine secretions in conventional T cells are barely detectable (Fig. 7C), Therefore, reduced severity in Con A-induced hepatitis in the B7-deficient mice correlates to decreased IFN-γ by NKT cells.

To further confirm the defect of NKT population in B7 mutant mice, we adopted an in vitro model to test the reactivity of NKT cells from WT and B7KO mice to its exogenous ligand, α-Galactosylceramide (KRN7000). To exclude the effect of weak costimulatory signaling from B7 mutant antigen presenting cells [47], both purified CD4 T cells from WT and B7KO mice was stimulated in vitro with purified CD11c+ dendritic cells from WT mice. As shown in Fig. 7D, after 48hours co-culture with KRN7000, CD4 T cells from WT have a stronger response, as indicated by more IFN-γ and IL-4 secretion. Because KRN7000 can only be recognized by iVα14 NKT cell in CD4 population, this result provided direct evidence of lower number and/or functional defect of NKT cells in B7KO mice.

## Discussion

By comparing WT mice with those with targeted mutations of CD28 or B7-1/2, we have demonstrated a major contribution of B7-CD28 interaction in the generation of iVα14 NKT cells in the thymus. Two lines of evidence presented here suggest that B7-CD28 interaction is required for the expansion of more mature NK1.1^+^ iVα14 NKT subsets. First, the number of iVα14 NKT cells in young mice, which are predominantly NK1.1^−^, is unaffected by these mutations. Second, the overall number of NK1.1^−^ iVα14 NKT cell was largely unaffected in the mutant adult mice, while the NK1.1^+^ iVα14 NKT cells were drastically reduced. Since earlier studies demonstrated that NK1.1^−^ iVα14 NKT cells are the precursors of NK1.1^+^ iVα14 NKT [26], [27], our data indicated that B7-1/2-CD28 interaction is not required for the generation of NKT cell precursors. The co-stimulation-dependent expansion of mature NKT is largely due to a critical contribution of B7-CD28 interaction in proliferation of the NKT cells as the incorporation of BrdU into the NK1.1^+^ NKT cells was drastically reduced. However, the percentage of apoptotic cells was unchanged in the B7-1/2-deficient mice and only marginally increased in the CD28-deficient mice.

As a consequence of defective NKT maturation in the thymus, we observed a significant reduction of the mature NKT cells in the spleen and liver, the major residences of the NKT cells. Nevertheless, the defects in the periphery were somewhat less pronounced than what was observed in the thymus. Theoretically, this can be due to increased emigration from the thymus, increased survival and increased proliferation in the mutant mice. We have not observed increased proliferation of NKT in the periphery of the mutant mice. In fact, we have observed decreased proliferation of NKT in the mutant mice. However, we have observed a decreased apoptosis of NKT cells in the spleens of B7-deficient mice. A similar trend was also observed in the CD28-deficient mice. The increased survival explains the lack of 1∶1 correlation between the reduction in the thymus and that in the spleen and liver. Another unexplained observation is the more severe phenotype observed in CD28-deficient mice as compared to B7-deficient mice. Since B7-1/2 interacts with both CD28 and CTLA-4, it is theoretically possible to explain the discrepancies between B7-1/2-deficient mice and CD28-deficient mice by invoking an opposite function of these two receptors. The fact that the deficiency was observed in older mice and that the CTLA-4-deficient mice do not survive to adulthood have made it difficult to test this hypothesis.

Concanavalin A–induced (ConA-induced) hepatitis in mice is a well-characterized model of T cell–mediated inflammatory liver disease. Recently, it has been demonstrated that invariant NKT (iNKT) cells play a key role in the development of ConA-induced hepatitis. Both J_α_18^−/−^ and CD1d^−/−^ mice that lack iNKT cells are resistant to ConA-induced hepatic injury [37], [38], [48]. In addition, the adoptive transfer of hepatic mononuclear cells from wild-type mice, but not from CD1-deficient mice, sensitized *gld* mice to Con A-induced hepatitis [37]. The later results demonstrated that NKT cell, other than other cell, is the major mediator of the disease process. Here we demonstrated that the B7-1/2- and CD28-deficient mice are more resist to the disease. In this model, IL-4 secreted by mainly NK1.1^−^ iVα14 NKT cells, was demonstrated to play a crucial role in causing NKT cells to express FasL and to contribute to Fas/FasL-mediated liver injury in an autocrine fashion [37], [38], [42]. Although our presented data appear to refute a role for IL-4 production by NKT cells, this is likely due to the fact that the data presented was obtained at 6 hours when the peak of IL-4 production had weaned [45], [46]. In addition, IFN-γ produced by NK1.1^+^ iVα14 NKT cells is also very important in mediating the disease process. IFN-γ can activate resident Kupffer cells and recruit macrophages to produce TNF-α, which subsequently causes liver injury [37], induces proliferation and cytotoxicity of NK cells [40] and regulates IL-4 signaling as well as its own signaling through induced suppressor of cytokine signaling (SOCS-1) [41]. Thus, the significant reduction of NK1.1^+^ iVα14 NKT cells may alleviate Con A-induced hepatitis in B7- and CD28- deficient mice by abrogating the production of IFN-γ. This hypothesis is supported by our analysis of ex vivo NKT cells, which revealed reduced IFN-γ producing cells following in vivo Con A stimulation.

Costimulatory pathways were traditionally viewed as critical modulators for the induction of T cell responses in peripheral lymphoid organs [49]. Subsequent studies in tumor [5]–[7] and autoimmune disease [8] models have revealed a critical contribution to the effector phase of T-cell immunity. More recent studies expand its contribution to differentiation of immature thymocytes and negative selection of T cells [10], [50]. In addition, the function of costimulatory molecules may extend beyond adaptive immunity, as several groups have suggested a role for the activation of NK cells [11]. Our data presented here demonstrate a role for T cell costimulation in both the development and function of NKT cells, which is widely regarded as a bridge between adaptive and innate immunity. Taken together, it is possible that costimulatory molecules modulate all major branches of cell-mediated immunity, including T cells, NK cells and NKT cells.

## Materials and Methods

### Experimental animals

Wild-type C57BL/6j, B7-1/2 double knockout and CD28 knockout mice on C57BL/6j background were purchased from the Jackson Laboratory (Bar Harbor, ME, USA). Different strains of Mice were maintained in the University Laboratory Animal Research Facility at The Ohio State University and The University of Michigan under specific-pathogen-free conditions. All animal experimental procedures were reviewed and approved by The Ohio State University and University of Michigan Institutional Animal Care and Use Committees.

### Cell Preparation

Single cell suspensions from the thymus and spleen were prepared by mechanical disruption in cold, serum free RPMI 1640 medium. The liver was perfused with 1xPBS via portal vein. Then, the liver fragments were incubated with 1mg/ml Collagenase type IV (Sigma, C5138) in 10mM Hepes-NaOH buffer (150 mM NaCl, 5 mM KCl, 1 mM MgCl_2_ and 1.8 mM CaCl_2_, pH 7.4) for 1 h at 37°C and gently dissociated. The whole material was passed through a syringe several times to get a single cell suspension. The liver mononuclear cells were recovered from the interface of 40% and 60% percoll after centrifugation. Red blood cells from single cell suspensions were removed by brief hypotonic lysis before cell surface staining.

### Antibodies and flow cytometry

Both cell surface markers and intracellular staining were analyzed by flow cytometry (Becton Dickinson, Mountain View, CA). The fluorescein-conjugated antibodies anti-CD4 (GK1.5), anti-CD8 (53-6.7), anti-TCR β chain (H57-597), anti-CD44 (IM7), anti-CD25 (PC61) and anti-NK1.1 (PK136) were purchased from BD PharMingen (San Diego, CA, USA). Phycoerythorin-conjugated α-GalCer loaded CD1d tetramer was prepared as described [28], [36]. The fixation and permeabilization solution kit (Cytofix/Cytoperm™, BD Pharmingen) was used for intracellular staining according to the manufacturer's protocol. The samples were analyzed by flow cytometry. Data acquisition was performed on a FACSCalibur instrument with CELLQUEST software (Biosciences, Mountain View, CA), and data were analyzed using FLOWJO software (Tree Star, Inc., Ashland, OR).

### Cell death and proliferation assay

The apoptotic lymphocytes were determined by their binding to Annexin V. After cell-surface staining, the cells were resuspended in Annexin V (BD PharMingen), dissolved in 10 mM Hepes, 140mM NaCl, 2.5mM CaCl_2_ and stained at room temperature for 15 minutes and then analyzed within an hour. To measure the proliferation of lymphocytes in vivo, mice were injected intraperitoneally (i.p.) with BrdU (1 mg/mouse in 100 µl PBS). Four hours later, the mice were sacrificed and single lymphocytes were prepared. BrdU incorporation was detected by flow cytometry with a BrdU Flow Kit, as described by manufacturer (BD PharMingen).

### Induction of ConA induced hepatitis

Con A (Sigma, C0412) was dissolved in pyrogen-free PBS and i.v. injected into mice through the tail vein at a dose of 20mg/kg. Sera from individual mice were obtained 16 h after Con A injection. Alanine aminotransferase (ALT) and aspartate aminotransferase (AST) activities were measured by the standard photometric method using Hitachi type 911 automatic analyzer (Tokyo). 25mg/kg Con A was used for short-term stimulation, and the splenocytes and hepatocytes were harvested 6 hours later for intracellular cytokine staining.

### Histological Examination

The livers from Con A treated mice were harvested after 16 h and then fixed in 10% formalin, embedded in paraffin, sectioned and stained with hematoxylin and eosin for histological examination. Specimens were examined under a light microscope. 3–4 images were collected from each section and the injured area was measured with MCID Analysis 7.0 (Imaging Research Inc., Ontario, Canada).

### In vitro stimulation of purified CD4 cells with α-Galactosylceramide

Spleens were harvested from 10–12 weeks old WT C57BL/6 and B7-1/2 mutant mice. Single cell suspension were treated for 45 min with 400 U ml^−1^ collagenase type IV and 50 µg ml^−1^ DNase I (Boehringer, Mannheim, Germany) before were incubated with anti-CD11c-coated magnetic beads. CD11c^+^ cells were positively selected according to the manufacturer's procedure **(**Miltenyi Biotec, Bergisch-Gladbach, Germany). Then all the flow-through was depleted with antibody mixture containing rat monoclonal antibodies against mouse CD45R (B220), CD11b (Mac1), CD16/32(2.4G2), CD11c (HB224) and CD8 (TIB210) according to manufacturer's manual (DYNAL, Oslo, Norway). Cell suspensions contained about 90% of CD11c^+^ cells and CD4^+^ cells respectively. After purification, 7×10^5^ CD4 cells from either WT C57BL/6 or B7-1/2 mutant mice were co-cultured with 3×105 CD11c^+^ cells in 1ml Bruff's medium containing different concentration of α-Galactosylceramide (KRN7000). 48 hours later, the supernatant was harvest for the detection of secreted INF-γ and IL-4 with ELISA. The relative amounts of cytokines were shown by absorbance read at 490nm (OD490nm).

### Statistical Analysis

Data were statistically analyzed with two-tailed student T-test. P<0.05 is significant and p<0.01 is highly significant.
